# Ways of coping among women with infertility undergoing assisted reproductive technologies in Ghana

**DOI:** 10.11604/pamj.2022.41.29.31319

**Published:** 2022-01-12

**Authors:** Josephine Mpomaa Kyei, Adom Manu, Duah Dwomoh, Agnes Millicent Kotoh, Kofi Agyabeng, Augustine Ankomah

**Affiliations:** 1 School of Nursing and Midwifery, College of Health Sciences, University of Ghana, Legon, Accra, Ghana,; 2 Department of Population, Family and Reproductive Health, School of Public Health, College of Health Sciences, University of Ghana, Legon, Accra, Ghana,; 3 Department of Biostatistics, School of Public Health, College of Health Sciences, University of Ghana, Legon, Accra, Ghana,; 4 Population Council, Yiyiwa Drive, Accra, Ghana

**Keywords:** Infertility, assisted reproduction technology (ART), coping, women, Ghana

## Abstract

**Introduction:**

infertility remains a public health challenge, especially among women in low- and middle-income countries. Assisted reproductive technologies (ART) provide effective remedies to infertility problems. Despite the use of these technologies in many countries, not much empirical studies have examined the coping strategies infertile women accessing ART adopt. We sought to explore various coping mechanisms used by women with infertility.

**Methods:**

a cross-sectional survey was conducted in five selected fertility centers in Accra. Overall, 150 women undergoing ARTs were consecutively sampled. The ways of coping questionnaire was used. Chi-square test and binary logistic regression model were used in testing for association between sociodemographic characteristics of women receiving ART and the use of coping strategies at 5% significance level.

**Results:**

the mean age of the respondents was 38.2 ± 6.1 years with 56% having completed tertiary education and 40% had been experiencing infertility for less than five years. All 46.7% of the respondents were in phase two of the treatment process. The most widely used coping strategy was positive reappraisal, with confrontational being the least form of coping strategy used. Age and educational level significantly associated with problem solving and positive reappraisal, respectively.

**Conclusion:**

the study concludes that women use various coping strategies whiles seeking ARTs. Women who were advanced in age and had attained higher level of education coped better whiles seeking ARTs. Those who had experienced infertility for a long period were able to cope through avoidance of difficulty situations and accepting the reality of the challenges. It can therefore be recommended that the coping strategy used among different subgroups of individuals that are seeking ARTs needs to be identified and women educated on whiles seeking ARTs.

## Introduction

Infertility remains a global public health challenge, especially among women in low- and middle-income countries [1]. Evidently, one in every six couples will face issues with infertility during their reproductive age [2]. Infertility has been found to cause many problems, especially in Africa where considerable value is placed on childbearing during adulthood [3]. It has been found to affect the physical, psychological, and social wellbeing of the individuals [4], these include the experience of anxiety, depression, divorce, discrimination, social isolation, lack of economic security and stigmatization [5,6]. In some communities, men and women are denied proper burial after death due to their failure to bear children [7]. Various degrees of economic burden such as experiences of poverty by individuals with infertility issues in their old age has also been identified [8]. This drives couples to look for interventions to enable them to achieve their reproductive aspirations.

To advance the treatment of infertility, Assisted Reproductive Technologies (ART), a procedure of establishing pregnancy by invitro handling of both human oocyst and sperm, or of embryos have come to the fore [2]. The ART process is found to be multidimensional and requires strict adherence to the steps involved to achieve a successful outcome [2]. The ARTs cycles present challenges that result in stress as well as psychological and emotional difficulties to individuals and couples going through the process [9,10]. These challenges have been implicated in the reasons for discontinuation of fertility treatment [11]. The experiences and responses of couples and individuals going through the ART process may vary according to the sociodemographic characteristics of the individuals and the phase/stage of the treatment. As noted by Gourounti *et al*. [10] the ability to cope with stress and challenges associated with the ART process is critical to ensuring a successful outcome. Studies on coping and coping strategies used by individuals undergoing ART are limited. Additionally, little is known about how individuals undergoing ART cope with the emotional, psychological and the economic burden associated with ART.

The possible difference among various subgroups as relates to age, educational status, the duration of infertility and the phase of treatment and the use of coping strategies remain largely unknown. There is therefore the need for a systematic investigation into how individuals undergoing the ARTs cope with the process. This is particularly important in Ghana and other African countries where children are highly cherished and celebrated and the societal pressure to have children is extremely intense [3]. Knowledge on this will assist in the design of appropriate interventions to strengthen the coping mechanisms of individuals seeking ART. The current study was thus conducted to elucidate how individuals cope with the ART in the Ghanaian setting, to address the gap in knowledge in ARTs and coping strategies.

## Methods

**Study design:** this was a facility based cross-sectional study. It involved the collection of data during a single period without a follow up. The study also focused on the outcome and the exposures in the participants at the same time.

**Study settings:** the study was conducted in some selected private fertility centres in the Greater Accra Region. These facilities were selected purposely because they provide various forms of ART services such as invitro fertilization (IVF), intra cytoplasmic sperm injection (ICSI), intrauterine insemination (IUI), sperm donation, embryo donation, oocyte donation, sperm freezing, oocyte freezing, embryo freezing and comprehensive semen analysis.

**Population:** the respondents for the study were women undergoing ART and were at various phases of the treatment. The step of ART involves three phases: phase I involve clinical assessment of clients before they are assigned to different ART methods. Phase II involves actual treatment process, which includes hormonal treatment and embryo transfer, while phase III comprises of clients who have successfully achieved pregnancy [9,10].

**Sampling and sample size determination:** this study focused primarily on women, because they go through the three phases of the ART, and they were better positioned to provide information on how they cope with the process. One hundred and fifty women who were undergoing ART were recruited consecutively over a period of nine months, beginning from 1^st^ May 2017 to 31^st^ January, 2018. Selected participants were administered questionnaire by the interviewers after signing the informed consent.

**Data collection:** a structured questionnaire was used to elicit respondents´ socio-demographic characteristics in addition to the Ways of Coping (WOC) tool developed by Lazarus and Folkman [12], to assess how the women coped with the ART processes. Although the Ways of Coping (WOC) compose of 66 items measuring 8 domains of coping strategies, 50 out of them were deemed culturally appropriate and were used in the current study. Each item was rated on a four-point Likert scale (1 - not used, 2 - somewhat used, 3 - used quite a bit, a 4 - used a great deal). The modified WOC tool was internally reliable with an overall Cronbach´s alpha of the WOC was 0.79. The 50 items were mapped onto eight subscales of coping strategies/domains, namely: confrontational (6 items), distancing (7 items), self-control (6 items), social support (6 items), acceptance-responsibility (4 items), escape-avoidance (8 items), problem solving (6 items) and positive-reappraisal coping (7 items) - supplementary material. The total score for each of the eight domains was obtained by summing the Likert-scale responses of the individual Likert-typed items that define that subscale. Higher scores indicate that a particular coping mechanism was used more often and vice versa. The total scores for each domain were dichotomized into “most used - 1” if the total score is greater than or equal to 50% of the total score for the domain under consideration otherwise “less used - 0”.

**Study variables:** the dependent variable was the levels of coping that was measured as a composite variable. The independent variables included the various phases of treatment that included phase 1, phase 2, and phase 3. The other independent variables included socio-demographic factors such as age, sex, ethnicity, religion, residence, and family background, level of income, marital status, and occupation.

**Data analysis:** data entry of completed questionnaire was entered into Microsoft Excel and imported into STATA version 15 for analysis. Frequencies and percentages were reported as summary statistics for categorical variables. Prevalence of each coping strategy was reported in terms of percentages and their corresponding 95% confidence intervals using the binomial exact estimates. Chi-square test of association and binary logistic regression were used in assessing the relationship and effect of sociodemographic characteristics of women receiving ARTs and the use of coping strategies. All statistical tests were carried out at 5% significance level.

**Ethical consideration:** ethical approval for the study was obtained from the Ghana Health Service Ethics Review Committee (protocol number: GHS/REC: 02/01/2017). In addition, institutional permission was received from the various facilities selected for data collection. Informed consent was obtained from the study participants. This was done after explaining the purpose of the study including the risk and benefits to the respondents. Participants were given the opportunity to ask questions before the written consent form was signed. Confidentiality as well as anonymity of the study respondents was ensured. Data was protected by putting filled questionnaire under lock and key and only accessible to the lead researcher. Each questionnaire had a code for easy identification and retrieval where necessary. A detailed labeling of variables and record names was ensured to avoid confusion.

## Results

**Sociodemographic characteristics of respondents:** a total of 150 respondents completed the questionnaire with a mean age of 38.2 ± 6.1 years. The most represented age group was 30 - 39 years (53.3%). More than half of the respondents (56%) had completed tertiary education. Mean duration of infertility was 6.7 ± 4.7 years, and about 40% had been experiencing infertility for less than five years while about a quarter (24.7) had undergone it for more than ten years. With regards to treatment phases, nearly half 46.7% of the women were in phase II of their treatment process (Table 1).

**Table 1:** sociodemographic characteristics of respondents

	Frequency	Percent
**Age in years**		
Mean (± SD)	38.2 ± 6.1	
<30	9	6.00
30 - 39	80	53.33
≥40	61	40.67
**Level of education**		
No education	2	1.33
Primary/JHS/middle school	33	22.00
Secondary/vocational	31	20.67
University/polytechnic	84	56.00
**Duration of infertility (in years)**		
Mean (± SD)	6.7 ± 4.7	
<5	60	40.00
5 - 9	53	35.33
10 - 14	23	15.33
>=15	14	9.33
**Treatment phase**		
Phase I	55	36.67
Phase II	70	46.67
Phase III	25	16.67

SD: standard deviation; JHS: junior high school

**Coping strategies used by respondents:** Table 2 presents the prevalence of coping strategies used by the women receiving fertility treatment with their corresponding 95% confidence interval. The most widely used coping strategy by the respondents was positive reappraisal (97.3%), followed by escape avoidance (96.7%). The least commonly used coping strategy was acceptance responsibility (46%).

**Table 2:** coping strategies used by respondents

Coping domain	Total domain score: mean ± SD	Percentage of people who “most used” a domain	95% CI
Confrontational	13.31 ± 2.70	71.33	61.99 - 77.20
Distancing	16.43 ± 4.21	72.67	64.80 - 79.62
Self-control	14.97 ± 3.37	90.67	84.84 - 94.80
Social support	17.93 ± 5.20	87.33	80.93 - 92.20
Acceptance responsibility	8.17 ± 3.28	46.00	37.84 - 54.32
Escape avoidance	18.67 ± 3.14	96.67	92.39 - 98.91
Problem solving	18.39 ± 3.14	87.33	80.93 - 92.20
Positive reappraisal	20.05 ± 3.45	97.32	93.31 - 99.27

SD: standard deviation

**Distribution of coping strategies versus sociodemographic characteristics respondents:** Table 3 shows the distribution of the usage of various coping strategies as related to various demographic characteristics of the respondents. In relation to age, all respondents who were less than 30 years used strategies in the self-control domain (100%) as a coping strategy and the least coping strategy used by them was acceptance responsibility (44.4%). On the other hand, respondents aged 30-39 years mostly used escape avoidance (97.5%) as a coping strategy and the least used coping strategy in this subgroup was acceptance responsibility (43.8%). All respondents who were 40 years or older indicated the use of coping strategies in the positive reappraisal domain (100%) with acceptance responsibility as the least used coping strategy (49.2%). From the chi-square test, age was found to be significantly associated with the use of problem solving as a coping strategy (p value < 0.05) (Table 3). The level of education considered as a subgroup and its influence on the coping strategies used revealed that, those who had no education (n=2) used all the coping domains equally (100%). All respondents with primary level of education, used positive reappraisal (100%) with the least used coping strategy being acceptance responsibility (33.3%). Respondents who had secondary/vocational level education used escape avoidance domain (93.5%) predominantly and less likely to use acceptance responsibility domain (29%). Respondent who had obtained tertiary level of education all used positive reappraisal (100%) and acceptance responsibility (56%) as the least used coping strategy. Educational level of was found to be significantly associated with accepting responsibility as a coping strategy (p<0.01) (Table 3). From Table 4, the binary logistic regression model showed that compared to women with primary educational level, those with tertiary level of education had about 2.9 times higher odds of using accepting responsibility as a coping strategy (aOR: 2.86 95%CI: 1.16 - 7.01).

**Table 3:** distribution of coping strategies and sociodemographic characteristics of respondents

	Coping domain							
	Confrontational	Distance	Self-control	Social support	Accepting reappraisal	Escape avoidance	Problem solving	Positive reappraisal
	n (%)	n (%)	n (%)	n (%)	n (%)	n (%)	n (%)	n (%)
**Age**							*	
<30	6 (66.67)	6 (66.67)	9 (100)	6 (66.67)	4 (44.44)	8 (88.89)	7 (77.78)	8 (88.89)
30-39	59 (73.75)	60 (75)	73 (91.25)	71 (88.75)	35 (43.75)	78 (97.5)	66 (82.5)	77 (96.25)
≥40	40 (65.57)	43 (70.49)	54 (88.52)	54 (88.52)	30 (49.18)	59 (96.72)	58 (95.08)	61 (100)
**Educational level**					**			**
No education	2 (100)	2 (100)	2 (100)	2 (100)	2 (100)	2 (100)	2 (100)	2 (100)
Primary	28 (84.85)	23 (69.7)	30 (90.91)	30 (90.91)	11 (33.33)	32 (96.97)	29 (87.88)	33 (100)
Secondary	22 (70.97)	20 (64.52)	27 (87.1)	25 (80.65)	9 (29.03)	29 (93.55)	28 (90.32)	27 (87.1)
Tertiary	61 (72.62)	64 (76.19)	77 (91.67)	74 (88.1)	47 (55.95)	82 (97.62)	72 (85.71)	84 (100)
**Duration of infertility (yrs)**		*						
<5	38 (63.33)	50 (83.33)	57 (95)	52 (86.67)	28 (46.67)	58 (96.67)	51 (85)	57 (95)
5-9	37 (69.81)	38 (71.7)	48 (90.57)	48 (90.57)	20 (37.74)	51 (96.23)	46 (86.79)	52 (98.11)
10-14	20 (86.96)	14 (60.87)	18 (78.26)	19 (82.61)	12 (52.17)	22 (95.65)	20 (86.96)	23 (100)
≥15	12 (85.71)	7 (50)	13 (92.86)	12 (85.71)	9 (64.29)	14 (100)	14 (100)	14 (100)
**Phase/stage**								
Phase I	44 (80)	38 (69.09)	48 (87.27)	50 (90.91)	25 (45.45)	52 (94.55)	47 (85.45)	54 (98.18)
Phase II	53 (75.71)	50 (71.43)	66 (94.29)	59 (84.29)	33 (47.14)	69 (98.57)	60 (85.71)	67 (95.71)
Phase III	18 (72)	21 (84)	22 (88)	22 (88)	11 (44)	24 (96)	24 (96)	25 (100)

N: frequency; %: row percentage; *P-value <0.05; **P-value<0.01; ***P-value<0.001

**Table 4:** effect of sociodemographic characteristics of women receiving assisted reproduction technology treatment on the use of coping strategies

Coping domain						
	Confrontational	Distance	Self-control	Social support	Accepting resp	Problem solving
	aOR (95% CI)	aOR (95% CI)	aOR (95% CI)	aOR (95% CI)	aOR (95% CI)	aOR (95% CI)
**Age**						
<30	1	1	1	1	1	1
30-39	1.87 (0.33 - 10.75)	1.84 (0.34 - 9.8)	0.64 (0.14 - 2.81)	3.04 (0.55 - 16.79)	0.9 (0.19 - 4.2)	1.37 (0.22 - 8.71)
≥40	1.2 (0.18 - 7.84)	3 (0.49 - 18.2)	1(empty)	4.3 (0.6 - 30.72)	0.85 (0.17 - 4.36)	7.41 (0.74 - 73.97)
**Educational level**					**	
No education	1(empty)	1(empty)	1(empty)	1(empty)	1(empty)	1(empty)
Primary	1	1	1	1	1	1
Secondary	0.38 (0.08 - 1.81)	0.76 (0.24 - 2.4)	0.58 (0.11 - 3.09)	0.55 (0.11 - 2.65)	0.79 (0.26 - 2.41)	1.57 (0.27 - 9.04)
Tertiary	0.35 (0.09 - 1.37)	1.28 (0.47 - 3.48)	0.95 (0.2 - 4.42)	0.94 (0.23 - 3.91)	2.86 (1.16 - 7.01)	0.82 (0.21 - 3.15)
**Duration of infertility (yrs)**		*				
<5	1	1	1	1	1	1
5-9	1.32 (0.48 - 3.65)	0.39 (0.14 - 1.05)	0.6 (0.13 - 2.74)	0.97 (0.26 - 3.57)	0.64 (0.28 - 1.47)	0.96 (0.31 - 3.03)
10-14	2.9 (0.58 - 14.55)	0.21 (0.05 - 0.82)	0.17 (0.02 - 1.15)	0.42 (0.08 - 2.36)	1.33 (0.41 - 4.32)	0.39 (0.06 - 2.57)
≥15	3.14 (0.48 - 20.49)	0.1 (0.02 - 0.47)	0.73 (0.05 - 9.73)	0.53 (0.07 - 3.95)	2.64 (0.66 - 10.61)	1(empty)
**Phase/stage**						
Phase I	1	1	1	1	1	1
Phase II	0.92 (0.34 - 2.48)	0.9 (0.38 - 2.16)	2.02 (0.5 - 8.16)	0.48 (0.14 - 1.63)	0.86 (0.39 - 1.9)	1.23 (0.4 - 3.82)
Phase III	0.75 (0.21 - 2.65)	2.48 (0.65 - 9.44)	0.81 (0.17 - 3.94)	0.64 (0.13 - 3.26)	0.71 (0.25 - 2.06)	3.9 (0.43 - 35.16)

* P-value <0.05; **P-value<0.01; ***P-value<0.001; empty: empty due to perfect prediction

Considering the duration respondents had been diagnosed of infertility and the coping strategy used indicated that respondents diagnosed of infertility for less than five (5) years used escape avoidance domain mostly (96.7%) with the least used coping domain being acceptance responsibility (46.7%). Positive reappraisal coping domain (98.1%) and acceptance responsibility (37.7%) were the most and least used domains respectively by respondents diagnosed of infertility between 5-9 years. All respondents diagnosed of infertility of between 10-14 years, were found to be using positive reappraisal (100%) while the least used acceptance responsibility domain (52.2%). Respondents who were experiencing infertility for 15 years and above all used escaped avoidance, problems solving and positive reappraisal (100%). The least used coping domain by this category of respondents was distancing (50%). The use of distancing strategy was the only coping strategy significantly associated with duration of infertility (Table 3). From the adjusted binary logistic regression model, use of distancing as a coping strategy was negatively related with duration of infertility. Thus, the odds of using distancing coping strategy were 61%, 79%, and 90% lower among women who have been infertile for 5-9 years, 10-14 years, and ≥15 years compared to those who have been infertile for less than 5 years respectively (Table 4). In relation to the phase of treatment (stage) that the respondents were in, those in phase I predominantly used positive reappraisal domain (98.2%) and least acceptance responsibility domain (45.5%) as the coping strategy. Respondents in phase II indicated their predominant use of escape avoidance domain (98.6%) and least acceptance responsibility domain (47.1%). All respondents in phase III indicated that they used positive reappraisal (100%) with acceptance responsibility domain as the least coping strategy used (44%) (Table 3).

## Discussion

This study investigated the coping strategies used by women undergoing assisted reproductive treatment. Prevalence of the ways of coping as conceptualised by Folkman and Lazarus [12] was examined among women with infertility and undergoing ARTs in five fertility centers in Accra, Ghana. These include confrontational, distance, self-control, social support, acceptance responsibility, escape-avoidance, problem-solving, and positive reappraisal coping strategies. It was observed in this current study that majority of the respondents were between the ages of 30-39 years (94%). This indicates that ARTs are often sought by individuals in their mid and late reproductive age after trying to conceive for a period. This finding is consistent with a study that reported delays in seeking treatment [13] with the reasons for this to include beliefs about the aetiology of infertility, the hope of achieving natural pregnancy and exploration of other forms of infertility treatment including “hospital shopping”. A significant proportion (60%) of the respondents had been experiencing infertility for more than five years confirming that the consideration of using ARTs to solve the problem of infertility was considered late when clients encounter this challenge [14,15]. This reflected the relative advanced reproductive age of the respondents in this study and other studies [16].

As found in other studies, a higher level of education influences the seeking of ARTs [17]. This study found out that more than half of the respondents had completed tertiary education. This may be due to the complexity of the procedure and the need to understand the process of ARTs with tertiary education being an advantage. Most of the respondents were in phase two (actual treatment phase) of their treatment process. In a study in Ghana, it was found that clients often seen at the fertility centres were in phase two [9] as they were in the actual treatment phase that required their presence in the facilities and so were readily available for the study. Individuals who experience infertility, are often under stress because of mental and social challenges they go through. The stresses have a major effect on their mental and physical health [18-20]. Coping strategies are therefore conscious efforts to reduce the stressful situations. They are actions usually used to deal with those stressors [21]. The commonest coping strategy adopted by the respondents in this current study was predominantly positive reappraisal. This involved the use of prayer for divine help. Several participants indicated that they were able to overcome their infertility challenges by having a strong faith and trust in God to give them a child.

Religiosity plays a significant role in the life of most Ghanaians, and it appears to shape their lives, particularly, the meanings attached to situations and events. This is similar to reports in other studies among couples with infertility [18,22-24] whereby the respondent indicated the use of religious activities including prayer to cope with the challenges that comes with infertility treatment, this finding also corroborates a study conducted in Ghana by Donkor and Sandall (2009) who found that a higher proportion of their respondents believed that their hope and faith in God would answer their prayers to have a child at the appropriate time [23]. Further, in South Africa, respondents indicated that they had unwavering faith in God to conceive, despite their failed attempts of achieving pregnancy [25]. The second commonly used coping strategy by respondents was escape-avoidance which consists of efforts to escape or avoid a stressing agent and fantasize about the possible solution to the problem [12]. The use of escape avoidance found in this study has been previously reported in a study [26].

In contrast, Yazdani *et al*. [27] in their study in Iran, identified the less use of escape avoidance strategy as a coping strategy among the respondents as compared to findings of the current study. Perhaps women with infertility in Ghana may be under pressure to have a child and therefore avoids any stress that comes with treatment and aim at a positive solution to treatment. Social support coping strategy use was fourth out of the eight domains studied. The current study found out that the women seeking ARTs were supported by others as well as their spouses to go through the process in the light of possible failure of the ART. Some studies also found that clients supported themselves throughout the ART process at every stage of the treatment [26]. Social support has been noted to play a significant role in achieving success in ART [28]. This current study revealed that confrontational coping was one of the coping strategies less used by respondents. It ranked eighth on usage out of the nine coping strategies studied. Contrary to this study, Peterson *et al*. [26] reported in their study that women used proportionately greater amounts of confrontational coping strategies whiles seeking ART. In the Ghanaian context, women are considered as the main cause of the infertility problem and therefore tend to be docile and do not exhibit aggressive attitude towards solving the problem.

The results of the logistic regression analysis revealed that the most widely used coping strategy by the respondents was positive reappraisal. This finding therefore indicates the contribution of religiosity in the coping strategies of individuals seeking ART. Additionally, there was a significant association between positive reappraisal coping strategy and educational level. This finding therefore indicates the contribution of education in the coping strategies of individuals seeking ART in this study group. Participants who had attained tertiary level of education are knowledgeable about the successes and failures of ARTs in the light of their own peculiar clinical situation and therefore believed in God for a positive outcome of the intervention even when the odds are high. A study also found that positive reappraisal coping strategies was used by older women whiles seeking ARTs [28].

In this current study, it emerged that the longer the number of years the woman had been looking for a child, the less likely that confrontational coping strategy was adopted. This observation could be attributed to the fact that the long duration of looking for a child might have built immunity in them to resist any possible negative expectations. Use of distancing as a coping strategy was found to be negatively related with duration of infertility. After waiting for a long period with no pregnancy, once they decide to go ahead to seek help for themselves by way of ART they determine not be influenced by comments or perceptions that may bother on stigma thus distancing themselves. Similar study by Peterson [26] found distancing coping strategy among infertile women whiles seeking infertility treatment.

Compared to women with primary educational level, those with tertiary level of education used accepting responsibility as a coping strategy. A study in China which focused on Chinese women seeking ARTs revealed that, a number of them used acceptance coping strategies during the process [29]. The study is the first of its kind in Ghana, which explored coping strategies used by women seeking ARTs.

**Limitations:** the use of cross-sectional design in this study, limits the establishment of cause-and-effect relationship.

## Conclusion

Insight into the coping strategies utilized by women undergoing ARTs is pertinent not only to advance theoretical models but also to design strategies to maximize the outcome of the process. This study found that women undergoing ARTs use various coping strategies with positive reappraisal being the most used. Duration of infertility and educational level predict distancing and accepting responsibility strategy respectively. Age and educational level are associated with problem solving and positive appraisal strategies respectively. It can therefore be concluded that women undergoing ARTs, do not use similar coping strategies during the process of seeking treatment.

**Recommendation:** from the conclusion, it is however, recommended that there should be a policy to ensure education on coping strategies as part of the counseling process during Arts. The coping strategy being used by the women seeking treatment, should be identified, and strengthened. The fertility hospitals should take women through new coping strategies, as this may be useful depending in making their treatment successful. Coping strategy used among different subgroups of individuals that are seeking ARTs need to be investigated further.

### What is known about this topic

- Demography of women undergoing ART is documented;
- The challenges that woman go through whiles seeking ART has also been reported on.

### What this study adds

- Women undergoing ART use coping strategies to mitigate the challenges they experience while undergoing treatment;
- This study found out that women undergoing ARTs mostly use positive reappraisal coping strategy.
